# An Optofluidic Temperature Probe

**DOI:** 10.3390/s130404289

**Published:** 2013-03-28

**Authors:** Ilona Węgrzyn, Alar Ainla, Gavin David Michael Jeffries, Aldo Jesorka

**Affiliations:** Department of Chemical and Biological Engineering, Chalmers University of Technology, Kemivägen 10, Göteborg SE-412 96, Sweden; E-Mails: ilona.wegrzyn@chalmers.se (I.W.); ainla@chalmers.se (A.A.); jeffries@chalmers.se (G.D.M.J.)

**Keywords:** Rhodamine B, Rhodamine 6G, multifunctional pipette, microfluidic device, microthermometer, temperature sensing, semi-contact, optofluidic, TRPV1

## Abstract

We report the application of a microfluidic device for semi-contact temperature measurement in picoliter volumes of aqueous media. Our device, a freely positionable multifunctional pipette, operates by a hydrodynamic confinement principle, *i.e.*, by creating a virtual flow cell of micrometer dimensions within a greater aqueous volume. We utilized two fluorescent rhodamines, which exhibit different fluorescent responses with temperature, and made ratiometric intensity measurements. The temperature dependence of the intensity ratio was calibrated and used in a model study of the thermal activation of TRPV1 ion channels expressed in Chinese hamster ovary cells. Our approach represents a practical and robust solution to the specific problem of measuring temperature in biological experiments *in vitro*, involving highly localized heat generation, for example with an IR-B laser.

## 1. Introduction

Accurate temperature determination is a key issue in many experimental studies within the fields of physics, chemistry and biology. The progressive miniaturization in many experimental methodologies has consequently also affected the instrumentation for thermal measurements. In particular the fast-developing research field of microfluidics, which is largely transdisciplinary and adds a strong engineering component to scientific investigations, has produced quite a variety of thermometry implementations within microfluidic chip technology. Many applications involving microfluidic chips depend on temperature measurement and control, mostly performed on flows confined to fluidic channels of micro- to millimeter dimensions. Examples include on-chip PCR amplification of DNA [1], separation methods based on thermal gradients [2,3], and devices enabling the investigation of kinetics and thermodynamics of chemical and biochemical reactions on the microscale [4,5]. However, local temperature measurement of biological samples in aqueous environments not confined to channels is challenging, in particular around single cells in cultures and tissue during microscopy experiments.

The range of techniques for temperature determination is extensive, with their physical foundations in thermoelectricity, temperature dependent resistance changes of electrical conductors, and optical phenomena such as fluorescence [6]. The majority of them, for example thermal imaging and a variety of spectroscopic methods, lack the spatial resolution to be applied in small scale devices. In microdevice technology, common methodologies to measure temperatures at small size scales, rely on the application of solid state sensing devices such as thermistors [7], resistance temperature detectors (RTD) [8,9], and thermocouples [10–13]. A number of other methods suitable for the microscopic size scale exist, most notably ion conductometry through a narrow glass capillary [14] or fluorescence based techniques, which either use strongly thermally-responsive fluorophores [15–17], or molecular beacons [18]. Thermistors as semiconductor resistance thermometers, and RTDs allow for high precision measurement, excellent stability and repeatability, but require elaborate fabrication. Microthermocouples also offer noteworthy advantages, namely short response time, simple construction and low production cost. They give moderately precise results, but the interface junction potentials require compensation. For integration into the surface of microfluidic chips and flow chambers, where the sensor is isolated from the aqueous flow to be investigated, such sensors can be conveniently obtained from commercial sources, or alternatively, directly fabricated into the device. Apart from the fabrication effort and other difficulties, these assemblies feature only limited positional flexibility, and microheating devices have to be designed so that heat source and sensor are co-localized. If more flexible local measurements are needed, *i.e.*, to bring the probe to the heat source, specifically designed microprobes need to be employed, which require custom fabrication, packaging and physical isolation from the aqueous medium. We have previously utilized such microthermocouple probes in combination with micromanipulation in various temperature studies on giant vesicles and in single cell electrophysiology [10].

Measuring the temperature dependence of ion conductivity using microcapillaries is an alternative means to measure the temperature locally in a microscale fluid environment. Glass needles benefit from their small footprint, and from the possibility to be specifically manipulated to a desired location in the sample [17]. A disadvantage is their fragility, which is particularly troublesome when measurements close to the surface, for example in a cell culture or on supported lipid films, need to be performed. They also require calibration individually for each fabricated needle, due to small variances in the tip geometry. Moreover, sensitive electronics are required to amplify the small signals generated by this probe, and the capillaries with their tiny orifices are prone to blockage. The benefits of glass needles are the relatively simple preparation, coupled with the ability to make fast and highly localized measurements.

A good substitute for solid state sensors, to be applied in microchannels within microfluidics devices, is the semi-invasive approach of utilizing an optical, fluorescence-based sensing principle [15,16,19,20]. The temperature is determined by following the change in fluorescence intensity of the fluorophore via a previously obtained calibration model [21]. Many interesting variants have been reported, for example the use of ratiometric dual-emission-wavelength measurements using molecular beacons in order to create a thermometer whereby the response is independent of the fluorophore concentration, as well as slight run-to-run changes in the experimental setup [18].

Many microscopy experiments in the life sciences utilize fluorescence or confocal imaging as standard techniques, thus the additional fluorescence intensity measurements for such a thermosensor does not pose any additional challenge or expense. Such systems also exhibit disadvantages, including the absorption of the fluorescent dye by the channel walls, most notable in polydimethylsiloxane (PDMS) based microfluidic devices, which hinders accurate fluorescence measurement, or the degradation of the fluorophore by photobleaching [19,22]. While the primary benefits of the optical readout are striking, with no need for electrical shielding or insulation, and lower fabrication and packaging requirements, it seems apparent that the use of dye solutions is limited to in-channel measurements.

**Figure 1.**
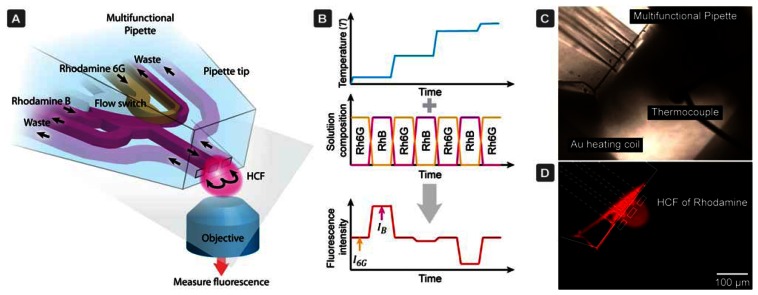
Concept of the microfluidic temperature probe with optical readout. (**A**) Schematic perspective view of the multifunctional pipette, which generates a hydrodynamically confined flow (HCF) in the environment where the temperature is probed. The content of this virtual flow cell can be quickly multiplexed between the strongly and weakly temperature responsive dyes. (**B**) The fluorescence signal of Rhodamine B (RhB) I_B_ rapidly drops with increasing temperature, while the fluorescence intensity of Rhodamine 6G (Rh6G) I_6G_ is only slightly influenced by temperature change. Outlined within is the concept of coupling the alternation of a change in temperature. (**C**) Micrograph of the multifunctional pipette in the calibration setup on top of a surface printed thin film heater (dark areas in left and right bottom corners) and a type E microthermocouple as a reference sensor. (**D**) Fluorescence image of the rhodamine HCF generated at the tip of the pipette (top down view). The diagonal line observed, is an artifact due to the pipette contacting the surface, which does not interfere with the flow or function.

We present here an open-volume and fluorescence based temperature measurement technique utilizing two rhodamine solutions in a hydrodynamically confined flow (HCF) device (Figure 1). By means of the previously reported multifunctional pipette (MFP) [23], a positionable open volume microfluidic device, we construct an optofluidic semi-contact thermometer which can be readily applied in microscopy experiments of biological and artificial cells, or tissue samples.

The multifunctional pipette primarily re-circulates a solution of the temperature-responsive fluorophore Rhodamine B (RhB) [24–27]. An aqueous solution of this dye is well known to exhibit an inverse dependency of its fluorescence emission intensity on temperature [28]. Using the solution switching capability of the device, we alternate RhB solution with Rhodamine 6G (Rh6G), which does not exhibit a dramatic dependence on temperature in the applicable range for biological systems. By making a comparative analysis of the ratio of fluorescence intensity obtained from either solution as the temperature is changed, we are able to exclude all environmental factors such as pipette position, heating source variances, microscope and detector settings (pinhole, signal gain *etc.*).

The main advantage of this concept is the avoidance of complex fabrication and interfacing steps, as is required for most solid state sensors, and utilize instead an established, readily available microfluidic device. We essentially redirect the fabrication challenge away from the sensor element, which involves fabrication, packaging, and interfacing, towards the simple replica molding procedure of a PDMS microfluidic chip. This greatly facilitates the construction of a microthermometer, and introduces a number of practical benefits. The device can be freely positioned close to the object of interest, and is at the same time unbreakable upon accidental contact with the surface. Most importantly, the specific features of the multifunctional pipette provide a remedy to some significant issues of fluorescence based temperature sensing: the virtual flow cell is unaffected by absorption of the dye to the device walls [29], and the fast exchange of rhodamine solutions counteracts fluorescence photobleaching, which has a detrimental effect on the intensity measurements. Due to the dual dye/single wavelength intensity ratio measurement, the device needs calibration only once for a given pair of dye concentration values used in the microfluidic devices.

## 2. Experimental Section

### 2.1. Multifunctional Pipette

The device used in the study was purchased from Avalance Biotech AB (Göteborg, Sweden). The fabrication of the multifunctional pipette has been described elsewhere [23]. If brief, a silicon wafer is coated with a phototpolymer SU-8, and patterned using standard photolithographic techniques. An outer structure frame (defining the pipette shape and the wells) is aligned to this wafer, forming both sides of the mold. Polydimethylsoloxane (PDMS) is then injected into the mold and cured. After cooling, the PDMS is removed, and oxygen plasma bonded to a 20 μm thick PDMS membrane. The outer shape, the wells and the tips are then cut, revealing the individual pipettes. These pipettes are finished by capping the bottom of the wells with an additional PDMS sheet, defining the volume and adding structural rigidity.

The key specifications of the pipette were as follows: channel dimensions: 30 × 30 μm, channel separation at the tip: 20 μm, distance from channels to base of the pipette tip: 20 μm, solution reservoir volume: 35 μL, on-chip switching capability: four independent solutions.

### 2.2. Chemicals

Rhodamine B and Rhodamine 6G were purchased from Sigma-Aldrich (Stockholm, Sweden). Stock solutions were prepared of both rhodamines, and the concentrations were adjusted so that the fluorescence emission intensities (543/554–604 nm) were approximately equal. Absorption spectra were measured in PBS buffer (pH 7.4), using 10 mm cuvettes. The absorbance values, which are convenient to reproduce the dye solutions (concentration ∼80 μM) used in the experiments, were 0.596 at λ_abs_max_ = 554 nm for for RhB, and 0.452 at λ_abs_max_ = 527 nm for Rh6B, respectively (Supplementary Information). 35 μL of each solution was transferred into the multifunctional pipette.

### 2.3. Microscopy

A laser scanning confocal microscope Leica IRE2 (Leica Microsystems GmbH, Wetzlar, Germany) equipped with a Leica TCS SP2 confocal scanner, and a long working distance objective 20×, NA 0.7, was used for data collection in the study. A HeNe laser provided the 543 nm, and an Ar/ArKr laser the 488 nm excitation wavelength. The 543 nm laser line was used to excite both the RhB and Rh6G solutions and the 488 nm laser to visualize the fluorescence of YO-PRO (emission collected between 500–535 nm) after activation of the human Transient Receptor Potential Vanilloid 1 (TRPV1) channels in Chinese hamster ovary (CHO) cells. Leica Lite (Leica Microsystems), Labview (National Instruments, Austin, TX, USA) and Matlab (Mathworks, Natick, MA, USA) packages were used to analyze the data.

### 2.4. Heating Systems

#### 2.4.1. Surface Printed Heaters

To generate a calibration curve, a non-optical microheating system in combination with a thermocouple reference sensor was used. The microheater consisted of a serpentine-shaped gold film, microfabricated onto a microscopy glass coverslip and coated with a layer of hard-baked SU-8 epoxy. The cover slip was interfaced using custom made microscope stage contacts. The microfabrication procedures (photolithography, film deposition, and epoxy-coating) have been previously described and applied by Markström et al. [10]. The reference temperatures for calibration have been determined using a Type E (chromel–constantan with 68 μV/°C) microthermocouple (Omega Engineering, Manchester, UK) connected to a custom built amplifier (10 mV/°C) with junction compensation, which was positioned closely to the micropipette channel exits. The thermocouple calibration measurements were performed in the range of 21-60 °C. The thermocouple readout was recorded after a stable temperature was achieved, while simultaneously recording a fluorescence image of the re-circulation zone from the micropipette.

#### 2.4.2. IR Laser and Optical Fiber

In the experiments, a 5W, 1470 nm (IR-B) semiconductor diode laser HHF-1470-6-95 (Seminex, Peabody, MA, USA) connected to 8A power supply (4308 LaserSource, Arroyo Instruments, San Luis Obispo, CA, USA) was used as an optical heat source. The laser light was transmitted to the measurement region by a 50 μm core diameter, 0.22 NA naked optical fiber (Ocean Optics, Dunedin, FL, USA). The polished end of the fiber was immersed into the aqueous sample solution, and micromanipulated to the region where the experiment was performed.

### 2.5. COMSOL Simulations

All simulations were performed with the finite element modeling software COMSOL 4.1 (COMSOL AB, Stockholm, Sweden) running on an Intel® Xeon® CPU (4 cores, 2.13 GHz) with 16 GB of installed memory. The model incorporated the physics of laminar flow (spf), transport of dilute species (chds) and heat transfer (ht/fluid), with approximately 200,000 elements in the mesh.

### 2.6. Thermal Activation of Human TRPV1 Channels in CHO Cells

Cells for expression of TRPV1 with the tetracycline regulated expression system (T-Rex) were received as a kind gift from Astra Zeneca R&D (CNS & Pain, Södertälje, Sweden). Before use, the cells were cultured for 2-6 days. The TRPV1 channel is a non-selective channel, permeable for cations, which contains six transmembrane domains [30,31]. It can be activated by several stimuli such as capsaicin, protons and temperatures higher than 42 °C [32].

A 1 μM solution of the carbocyanine nucleic acid dye YO-PRO^®^-1 (Life Technologies Ltd., Paisley, UK) in extracellular buffer (140 mM NaCl, 5 mM KCl, 1 mM MgCl_2_, 10 mM HEPES, 10 mM D-glucose, 10 mM Na_4_BAPTA, pH 7.4) was added to the culture dish containing the cells prior to the experiment, but could not enter the cell through the TRPV1 channels below the activation temperature. The fluorescence intensity of YO-PRO-1 increases significantly when it enters a cell and binds to DNA. For the ion channel activation/dye uptake experiment, the temperature was increased locally stepwise by means of the IR laser heater, and monitored with the pipette thermometer. When the desired temperature was reached, the fluorescence emission of YO-PRO-1 was recorded for the period of six minutes (five different temperature points were measured).

## 3. Theory and Model

The physics of the pipette thermometer is governed by the convection-diffusion equation. We consider a stationary measurement regime (after equilibration):

(1) $$\vec{v}\cdot \nabla c=D\nabla^{2}c$$

where *v* is the convective flow velocity, *D* is the diffusivity and *c* is the concentration of the dye. The concentration distribution depends on the Péclet number, a dimensionless scaling parameter: *Pé* = *νL*/*D.* The temperature distribution can be calculated by an equivalent equation, where the concentration is replaced by temperature *T*, and the diffusion by the thermal diffusivity *D_therm_* = 1.4 × 10^−7^ m^2^/s. The thermal diffusivity is about 380× higher than the molecular diffusivity of small molecules, for example, dye molecules such as rhodamine B or 6G. Another relevant difference between the thermal and molecular diffusivity lies in the boundary conditions. While molecular transport is confined to a liquid environment, the thermal diffusion also occurs at a nearly equal rate in solids, *i.e.*, heat dissipated through the glass bottom of the dish and pipette tip itself. The key to correct pipette function is to use a size scale, and flow parameters, so that *Pé_mol_* is sufficiently high to confine the dye and avoid contamination of the open volume, while *Pé_therm_* is sufficiently low to allow that the liquid in the confined volume equilibrates thermally with the surrounding, which is necessity for accurate thermal sensing. Another critical aspect is the determination of an operation regime which is least sensitive to experimental variances which may change from run to run, including the microscope settings, pipette position and angle *etc.*, in order to ensure repeatability and a universally valid calibration. For this we briefly describe how the fluorescence based intensity signal is actually formed.

Intensity signal *I* in microscope can be described as:

(2) $$I=\int_{V}P\cdot G\cdot A(\vec{r})c(\vec{r},\vec{v},D(T))f(T(\vec{r},\vec{v},T_{s}))d{\vec{r}}^{3}$$

where *P* is the power of the excitation light, *G* is the sensor gain. *A*(*r⃗*) is the spatial optical response function, describing how efficiently fluorescent light is gathered from different spatial locations. It depends on the pipette position, objective and focus. *c*(*r⃗, v⃗,D*(*T*)) describes the spatial concentration distribution of the dye, which depends on the flow *v⃗* and diffusivity *D*(*T*). *f*(*T(r⃗, v⃗*, *T_s_*)), describes the molecular fluorescence, which depends on the temperature *T*(*r⃗,v⃗,T_s_*), which further depends on the flow and the local temperature *T_s_*, which itself is of interest to be sensed. The fluorescence response is thus depending on a multitude of parameters, nearly impossible to reproduce and take into account fully. In order to simplify this situation, we have taken advantage of the switching function of the pipette, allowing to exchange between two similar fluorescent dyes, having similar diffusivity *D*. If we also maintain the flow rates *v⃗* during exchange, use the same microscope settings (focus, spot size *etc.*) and also same excitation and fluorescence measurement parameters, then *P, G, A, c* and *V* will be identical. In this case the ratio *I_B_*/*I_6G_* becomes:

(3) $$\frac{I_{B}}{I_{6G}}=\frac{\int_{V}A(\vec{r})c(\vec{r},\vec{v},D(T))f_{B}(T(\vec{r},\vec{v},T_{s}))d{\vec{r}}^{3}}{\int_{V}A(\vec{r})c(\vec{r},\vec{v},D(T))f_{6G}(T(\vec{r},\vec{v},T_{s}))d{\vec{r}}^{3}}$$

Since the contributions *f* are having a non-linear dependence on temperature, this equation is still too complex. However, if the flow rates are sufficiently low, such that the hydrodynamically confined volume becomes thermally equilibrated with the surrounding, then *T* = *T_s_*≠*f f*(*r⃗*), thus most of the integral components cancel out and Equation (3) becomes:

(4) $$\frac{I_{B}}{I_{6G}}=\frac{f_{B}(T_{s})}{f_{6G}(T_{s})}$$

where the ratio *f_B_*(*T_s_*)/*f_6G_*(*T_s_*) is solely a molecular property of the dyes, depending only on the temperature, but not on the device, microscope, sensor or illumination settings.

Therefore, this ratio is universal and an easily transferable calibration parameter. The only crucial aspect is to ensure the accuracy of concentration values of both fluorescent dyes, which can be easily determined by absorption spectrometry (Supplementary Information). One other assumption has to be made in this model: Only a small fraction of the excitation light is absorbed in the measured volume, *i.e.*, the measured volume is adequately illuminated. If *ε* ≈ 10^5^ M^−1^·cm^−1^ (Supplementary Figure), a maximum possible path length of *l* ≈ 30 μm, and a dye concentration *c* ≈ 100 μM, then the absorption of excitation light is less than 7%, which can be considered negligible.

### Simulation

A finite element model has been created to find suitable flow conditions, which would confine the dye molecules, while allowing for sufficient thermal equilibration. These simulations were also used for a simple perturbation analysis of the pipette, in order to determine how the signal can be affected by small deviations in flow rate *Q*, and outflow-inflow ratio *R*:

(5) $$\frac{\Delta I}{I}=\underset{a}{\underset{︸}{\frac{Q}{I}\cdot \frac{\partial I}{\partial Q}}}\frac{\Delta Q}{Q}+\underset{b}{\underset{︸}{\frac{R}{I}\cdot \frac{\partial I}{\partial R}}}\frac{\Delta R}{R}$$

**Figure 2.**
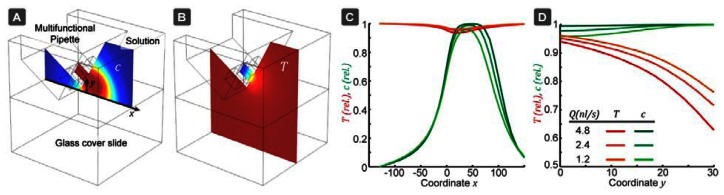
Finite element model of the pipette thermometer. (**A**) Hydrodynamic confinement of the fluorescent dye. (**B**) Thermal equilibration of the out-flowing solution. (**C**) Concentration and thermal equilibration profile along the bottom surface of the chamber (e.g. culture dish) and vertically in front of the pipette tip (**D**), for different flow rates Q. The graphs are normalized such that 0 corresponds to the surrounding liquid composition (C) and completely non-equilibrated temperature (T), while 1 corresponds to outflow composition and full thermal equilibration.

We have determined the first order perturbation coefficients *a* and *b*, which are important to estimate how slight pressure fluctuations and flow channel imbalances could affect the measurement result. The pipette has three channels of equal size, with a cross-section of 30 × 30 μm^2^ separated by a channel wall of 20 μm, as well as a 20 μm bottom membrane. The pipette is positioned at an angle of 30° to the horizontal and positioned 10 μm above the glass bottom. The glass bottom layer is 150 μm thick (#1 glass). A representation of the model and the results of the simulations can be seen in Figure 2. The outflow was set to *Q* = 1.2,2.4 and 4.8 nL/s and the inflow rate was adjusted such that *Q_out_*/*Q_in_* = 0.45, as used in the experiments. The simulation volume was 200 × 200 × 200 μm^3^ around the pipette tip. In a first step, the flow-field was calculated and the results were used for evaluation of the concentration profile and the thermal equilibration. The diffusivity of the rhodamines was *D*(20 °*C*) = 4.2 × 10^-10^ m^2^/s at room temperature, while at *D*(60 °*C*) = *2*.4*D*(20 °*C*) [33]. The thermal diffusivity, on the other hand, is only very slightly affected by temperature changes in this range (less than 1.1×).

From our simulations, the perturbation parameters found were *a* = 0.18 and *b* = 0.0006. Thermal equilibration near the surface is around ∼95% and through the entire volume ∼87% (Table S1 in Supplementary). However, the actual extent of thermal equilibration must be higher, since the pipette tip itself is also heat conductive (neglected in the above described model). The concentration drop due to diffusion is negligible ∼1%. These values define the maximum variations, which are possible due to slight changes in the pipette position. The parameters *a* and *b* are only weakly dependent on the flow rate and the *Q_out_/Q_in_* ratio, which indicates a stable calibration.

## 4. Results and Discussion

### 4.1. Calibration Curve

**Figure 3.**
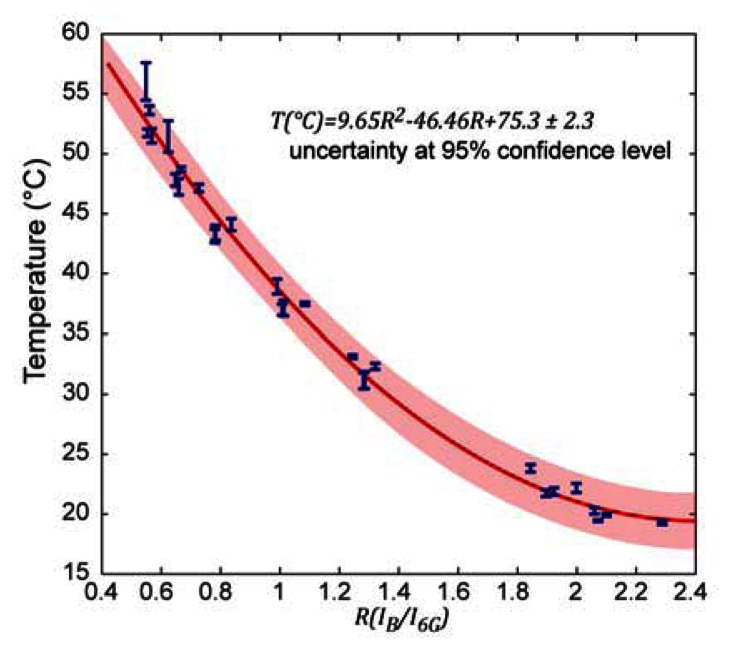
Calibration curve of pipette thermometer, showing the relation between fluorescence intensity ratio I_B_/I_6G_ and temperature T. Temperature dependent measurements were carried out in both directions (to account for a possible hysteresis), at two different flow rates and at two different distances of the channel outlets from the bottom plane. All error bars are given for the 95% confidence level.

After measuring the fluorescence intensities (single wavelength excited) of both dyes at different temperatures, a series of intensity ratios I_B_/I_6G_ could be calculated and plotted *vs.* temperature. The data points were fitted by a second order polynomial (Figure 3). The temperatures for the calibration model were determined using a calibrated thermocouple (Figure 1). The dynamic range of the sensor does not exceed the temperature range relevant for biological systems. It is likely that different dye pairs can be used to address other desired dynamic ranges and sensitivity.

This calibration curve is universally transferable to other experimental setups and conditions as long as the same dyes and concentration ratios are used in the actual measurements. It is not strictly required to replicate absolute concentrations, as long as the ratio is preserved and the fluorescence intensity change over the temperature range of interest is covered by the dynamic range of the optical fluorescence detector.

The calibration curve in Figure 3 features six discernible groups of data points, each of which corresponds to a set of measurements under varied experimental conditions. In each group alterations of either (a) the channel height above the bottom (0–10 μm), or (b) the flow rate (2.4 nL/s–4.8 nL/s), or (c) the direction of temperature change (up–down) were made in order to test the measurement repeatability, and thereby the validity of the theoretical model. These quite aggressive variations confirm, on one hand, independency from external influences, but are, on the other hand, the main cause of the somewhat decreased precision of the sensor. This calibration model, based on a single excitation wavelength ratiometric measurement, is universally transferable. Absolute temperature measurements can thus be performed on samples directly without re-calibration.

### 4.2. Thermal Activation of TRPV1 Channels in CHO Cells

**Figure 4.**
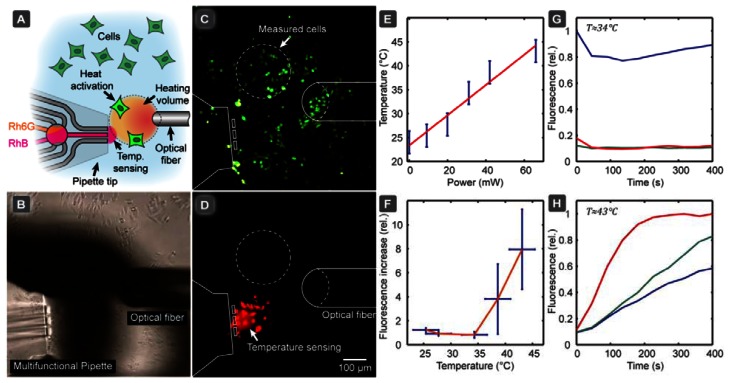
Heat activation of temperature sensitive ion-channels TRPV1, over-expressed in CHO cells, measured as a YO-PRO-1 uptake assay. (**A**) Schematic drawing of the concept of the experiment. Small groups of cells are activated by localized heating with IR-B radiation through the optical fiber (50 μm core). Ion-channel activation is characterized by the passage of the prefluorescent dye YO-PRO-1, while temperature is simultaneously probed using the pipette thermometer. (**B**) Brightfield micrograph of the actual experimental setup, showing the pipette and the optical fiber in close vicinity to the cells. (**C**) Fluorescence images of cells, measuring the intracellular fluorescence (λ_exc_ = 488 nm) in the region shown in (A). (**D**) Fluorescence image of the same region recorded in the rhodamine channel (λ_exc_ = 543 nm). The cells in the vicinity of the HCF zone appear brighter due to membrane absorption of rhodamine. (**E**) The equilibrium temperature depending on laser power. (**F**) The cellular fluorescence response depending on the temperature, measured as relative fluorescence increase. (**G**–**H**) Time course of the cellular fluorescence, after switching on the laser heater (G) below (≈34 °C) and (H) above (≈43 °C) the activation temperature. All error bars are given at the 95% confidence level.

We subsequently applied the calibration model in an application example, in which we investigated the thermal activation of heat-sensitive TRPV1 ion channels in single CHO cells. In order to determine the activation temperature of TRPV1 ion channels, which are over-expressed in the specific CHO cell line we employed, we used an IR-B diode laser coupled to an optical fiber. This served as a localized heat source to activate the ion channel of a selected group of cells, allowing the DNA binding stain YO-PRO-1 to enter the cell, as a reporter of activation (YO-PRO-1 uptake assay). Figure 4 shows the experimental setup (A, B), and the fluorescence image of the activated cells (C). Since the cells readily absorb rhodamine (Figure 4(D)), a region of interest in-between the cells was defined and used for temperature sensing. In Figure 4(D) the linear dependency of the equilibrium temperature from the laser power is presented, giving an indication which power range has to be approximately provided to cover the dynamic range of the pipette thermometer. A low power fiber-coupled diode laser is normally sufficient for this kind of investigation, but we note that coupling losses between fibers can significantly reduce the available power, depending on the core diameter of the receiving fiber.

Figure 4(F) depicts the relative YO-PRO-1 fluorescence increase depending on temperature, indicating that that TRPV1 activation occurred when the temperature exceeded 40 °C, which is in accordance with the literature. The calibration curve in Figure 3 was used to calculate the temperature values shown in Figure 4(F). A time course of the cellular fluorescence intensity change (relative), determined for three individual cells after switching on the IR-B laser, is presented before Figure 4(G) and after Figure 4(H) the activation temperature was reached. Note that the fluorescence intensities of the three cells are somewhat different, reflecting small differences, such as size, between the individual cells. The cells were selected such that the distance between the cells and the thermometer from the long (horizontal) axis of the laser fiber was approximately the same, in order to ensure that the measured temperature corresponds to the temperature the selected cells experience. Alternatively, the optical fiber can be repositioned for each cell to ensure the same distances selected cells, either by micromanipulating the fiber or translating the stage.

This exemplary study shows that the pipette thermometer can serve as a convenient auxiliary device to probe local temperatures under the conditions of single cell experiments, where closed channel microfluidic thermometers are unsuitable, solid state thermal sensors are less suitable (IR absorption, lack of positionability), and ion conductometry by means of glass capillary is risky (clogging risk by cell debris, proximity to surface). The accuracy of the temperature measurement achieved with this probe is sufficient to make semi-quantitative statements (above/below/close to activation temperature), which is often satisfactory for initial testing.

## 5. Conclusions

We have demonstrated the possibility of an open volume microfluidic thermometer, using a hydrodynamically confined flow device and optical fluorescence readout generated from a single excitation wavelength. Two fluorescent dyes with different thermal fluorescent responses are sequentially pumped through a virtual flow cell produced by the device. Assuming thermal equilibrium between the flow cell and the surrounding environment, and sufficiently low dye concentrations to ensure adequate illumination, the temperature can be determined directly by measuring the fluorescence intensity ratio between the two dyes in conjunction with an established calibration model. In contrast to most closed-channel microfluidic thermosensing devices with optical readout, our thermosensor is positionable, meaning that it can be brought very close to objects of interest. This allows for temperature measurements of biological experiments *in vitro* involving highly localized heat generation, for example by IR-B laser radiation. The optical detection principle is also beneficial in experiments involving sensitive electrical measurements, for example when patch clamp electrophysiology, electrochemical detection or impedance measurements are performed.

The multifunctional microfluidic pipette provides non-conventional opportunities to perform experiments that are otherwise difficult to perform. One example is the direct temperature measurement in the vicinity of a single adherent biological cell. Envisioned application areas are heat- or cold-activated ion channel studies, or other heat-assisted experiments on cells or tissue slices, in conjunction with confocal, or most beneficially, total internal reflection (TIRF) microscopy. We have shown that the calibration model is universally transferable, meaning that absolute temperature measurements are possible, independent from small day-to-day changes in the experimental conditions.

Future opportunities exist to improve some figures of merit, in particular the currently moderate precision (see Section 4.1) and the spatial resolution of the thermal sensor. In order to increase spatial resolution, the recirculation volume has to be decreased. This can in principle be achieved by decreasing the channel dimensions, but countermeasures against the increasing cross-boundary diffusion are required. One possible remedy is the use of fluorescent nanoparticles, for example, polymer nanospheres or vesicles loaded with the fluorescent dyes, so that smaller channels can be used at lower *Pé_therm_*. If such particles are carefully chosen such that they do not bind or otherwise interfere with the objects of interest, and absorb at a suitable wavelength, they might very well be routinely added to MFP superfusion protocols, which would provide a facile means of *in-situ* temperature measurement.

## Supplementary Material
