# Supplementary material for: An Optofluidic Temperature Probe
Source: Sensors (Basel). 2013 Mar 28;13(4):4289–302. doi: 10.3390/s130404289 (PMC3673084; doi:10.3390/s130404289)
Supplement: Supplementary file 1 [file sensors-13-04289-s001.pdf]

OPEN ACCESS

sensors

ISSN 1424-8220

www.mdpi.com/journal/sensors

Supplementary Information

An Optofluidic Temperature Probe. *Sensors* 2013, *13*, 4289-4302

Ilona Węgrzyn, Alar Ainla, Gavin David Michael Jeffries and Aldo Jesorka *

Department of Chemical and Biological Engineering, Chalmers University of Technology, Kemivägen 10, Göteborg SE-412 96, Sweden; E-Mails: ilona.wegrzyn@chalmers.se (I.W.); ainla@chalmers.se (A.A.); jeffries@chalmers.se (G.D.M.J.)

* Author to whom correspondence should be addressed; E-Mail: aldo@chalmers.se; Tel.: +46-31-772-6112; Fax: +46-31-772-6120.

Figure S1. UV-Vis absorption spectra of the RhB (blue) and Rh6G(red) solutions used in the study (sixteen times diluted).

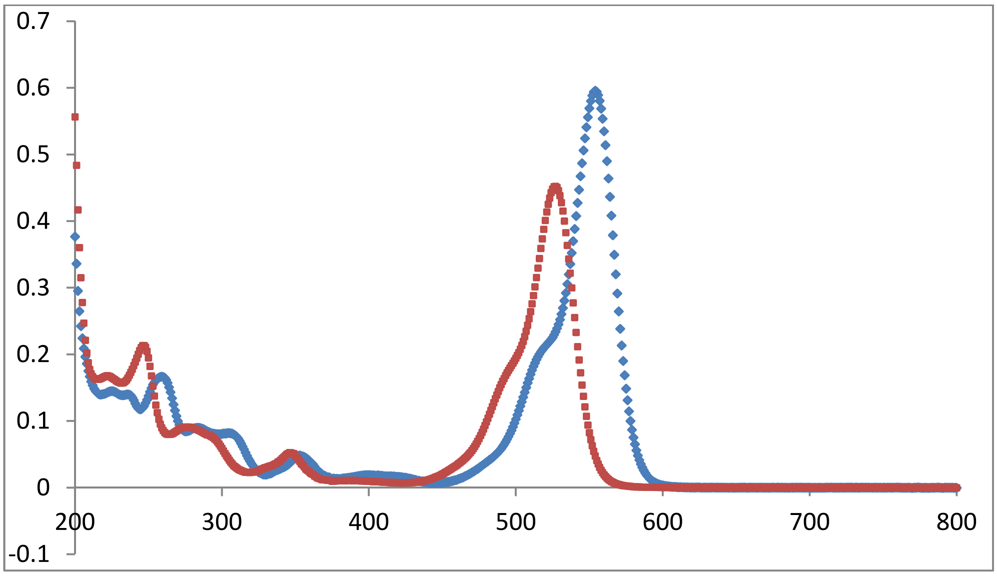

Dye concentrations calculated, using Lambert-Beer's Law:

RhB: Ext. Coeff. ε_{mol} = 106,000 $M^{-1}\cdot cm^{-1}$ ($\lambda_{abs, max}$ = 554 nm, A = 0.595675)

Concentration before dilution: 89.9 µM

Rh6G: Ext. Coeff. ε_{mol} = 116,000 $M^{-1}\cdot cm^{-1}$ ($\lambda_{abs, max}$ = 527 nm, A = 0.452295)

Concentration before dilution: 62.4 µM

The measurements were performed in water within 10 mm quartz cuvettes. The solutions used in the pipette thermometer were 16× diluted before absorption measurement. Note that the absolute concentration values are only approximate, since the literature extinction coefficients used in the calculation were determined in ethanol. Absolute concentrations are not required for calibration and temperature measurement, it is important that the concentration ratio of 1.44:1 (RhB:Rh6G) is established in order to use the calibration model described in the main text.

Table S1. Thermal equilibration and dye concentration depending on flow rate. The flow rate preferentially used in the experiments (2.4 nL/s) is highlighted in blue.

$Q\left(\frac{nL}{s}\right)$	$T_{surface}$	$T_{average}$	T_{min}	$c_{surface}$
1.2	96%	89%	76%	96%
2.4	95%	87%	71%	99%
4.8	94%	83%	63%	99.7%

© 2013 by the authors; licensee MDPI, Basel, Switzerland. This article is an open access article distributed under the terms and conditions of the Creative Commons Attribution license (http://creativecommons.org/licenses/by/3.0/).
